# Comparison of the analgesic effects of oxycodone vs. sufentanil on postoperative pain after laparoscopic gallbladder-preserving cholecystolithotomy: a prospective randomized controlled trial

**DOI:** 10.3389/fsurg.2024.1382759

**Published:** 2024-04-24

**Authors:** Ye Wang, Meng Wu, Lin Zhao, Xiaojian Yan, Lei Zhao

**Affiliations:** ^1^ Department of Anesthesiology, Peking University Shougang Hospital, Beijing, China; ^2^ Department of Anesthesiology, Xuanwu Hospital Affiliated with Capital Medical University, Beijing, China

**Keywords:** oxycodone, sufentanil, laparoscopic gallbladder-preserving cholecystolithotomy, inflammatory factors, postoperative pain

## Abstract

**Background:**

We aimed to compare the anesthesia induction effects of oxycodone and sufentanil on postoperative pain in patients undergoing laparoscopic gallbladder-preserving cholecystolithotomy, as well as changes in serum levels of inflammatory factors (TNF-α, IL-6, and IL-10) in the perioperative period.

**Methods:**

Sixty patients who underwent laparoscopic gallbladder-preserving cholecystolithotomy were evenly divided into oxycodone (O) and sufentanil (S) groups. In groups O and S, oxycodone (0.3 mg/kg) and sufentanil (0.3 ug/kg) were administered, respectively, followed by propofol (2 mg/kg) and rocuronium (0.6 mg/kg). In both groups, the intraoperative electroencephalography double-frequency index was used to guide the use of sedative and analgesic drugs, assessing the follow-up analgesic effect (VAS), degree of sedation (Ramsey), and postoperative complications at seven different time points (0, 0.5, 2, 4, 6, 8, and 24 h postoperatively).

**Results:**

Compared with the S group, patients in the O group exhibited lower VAS scores within 24 h postoperatively (*P* < 0.001), but there was no statistical difference between wound and shoulder pain scores (*P *> 0.05). Regarding postoperative awakening and extubation duration, O group patients experienced shorter times and better remedial analgesia (*P* < 0.05). In terms of the degree of sedation, the Ramsay score decreased at 0 h postoperatively compared with the S group (*P* < 0.001).

**Conclusion:**

Compared with sufentanil, oxycodone anesthesia induced better postoperative analgesia and less inflammatory responses in patients undergoing laparoscopic gallbladder-preserving cholecystolithotomy.

**Clinical Trial Registration:**

This study has been approved by the Ethics Committee of Peking University Shougang Hospital, with ethical approval (No. IRBK-2020-009), and has completed registration in the Chinese Clinical Trials Register (http://www.chictr.org.cn/) (ChiCTR2000031230).

## 1 Introduction

Along with the development of medical technology and optimization of medical equipment, laparoscopic gallbladder-preserving cholecystolithotomy (LGPC) has matured in recent years. This surgical approach not only preserves gallbladder function but also greatly reduces trauma, significantly improving the quality of life of patients after surgery. Thus, it has become one of the main clinical treatments for gallstones (1–4). Although this method has certain advantages such as quick recovery and a shorter hospital stay, 30%–70% of patients experience significant pain following the procedure leading to atelectasis and sympathetic hyperactivity (5, 6). In addition, postoperative pain is a key factor leading to the increased release of inflammatory cytokines such as tumor necrosis factor-α (TNF-α) and interleukin (IL)-6 and the diminished release of anti-inflammatory cytokines such as IL-10 in addition to the surgical trauma itself.

Optimal anesthesia induction and analgesia can effectively reduce the strong stress response triggered by sympathetic excitation, contribute to surgical recovery, significantly reduce the possibility of acute to chronic pain, increase patient comfort, and improve the postoperative quality of life of patients. Sufentanil has the advantages of rapid onset and few adverse reactions; however, its half-life is only 0.5 h (7). Postoperatively, patient-controlled intravenous analgesia (PCIA) usually requires continuous infusion, which may increase the risk of respiratory depression. Moreover, as *μ* receptor agonists, fentanyl and sufentanil have limited effects on *κ* receptors which are implicated in visceral pain. Therefore, despite the use of PCIA after abdominal surgery, vague, diffuse, and poorly defined discomfort remains widespread and is one of the main postoperative complaints in patients undergoing such procedures (8). As an emerging drug for inducing clinical anesthesia, oxycodone possesses a dual agonistic effect on *μ* and *κ* receptors, offering a more targeted approach compared to *μ* receptor agonists (9).

However, the nature and duration of postoperative pain after LGPC have not yet been clearly reported. At present, it is believed that it mainly consists of three parts: incision pain, visceral pain, and shoulder pain. Bile flow into the abdominal cavity caused by the operation may aggravate visceral pain and infection (1). The high clinical incidence of physical pain imposes a substantial burden on society; however, as attention gradually shifts toward visceral pain, it has been found that it causes an even greater social burden. In addition, most of the opioid analgesics used in clinical practice are classified as central µ-receptor agonists, and their analgesic efficacy in addressing somatic pain surpasses their effectiveness in alleviating visceral pain. As a new clinical drug with better efficacy in relieving visceral pain, oxycodone may fill this gap. Previous reports have shown that the analgesic effect of oxycodone is mainly exerted by its interactions with opioid receptors in the central nervous system and smooth muscles, and semisynthetic dual-receptor agonists have advantages in the treatment of visceral pain. Therefore, the purpose of this study was to clarify the effects of oxycodone induction on postoperative pain in LGPC along with the changes in perioperative serum levels of TNF-α, IL-6, and IL-10, with the aim of providing a clinical reference for the medication method and timing of oxycodone in this type of surgery.

## 2 Methods and analysis

### 2.1 Study design and subject allocation

This was a prospective, randomized, controlled, single-blind trial conducted at Peking University Shougang Hospital between July 2020 and January 2021.

All patients met strict inclusion criteria which were as follows: (1) aged 18–65 years; (2) scheduled to undergo elective laparoscopic cholecystostomy; (3) classified as American Society of Anesthesiologists (ASA) grade I; (4) possessing a body mass index (BMI) of 18–30 kg/m^2^; and (5) having voluntarily provided informed consent. The exclusion criteria were as follows: (1) presence of chronic pain; (2) presence of severe cardiovascular disease; (3) presence of severe respiratory disease or other severe systemic diseases; (4) history of digestive bleeding or peptic ulcer in the past 2 months; (5) dependence on alcohol or opioids; (6) long-term use of other psychotropic drugs; and (7) allergies to any of the drugs used in the institute. The elimination criteria comprised: (1) conversion of surgical method to cholecystectomy; (2) ineligibility for laparoscopic surgery; (3) history of cardiovascular and cerebrovascular accidents or prior surgical operation; and (4) specific conditions necessitating postoperative endotracheal intubation and transfer back to the intensive care unit.

Sixty patients were randomized into two groups using random numbers generated by computer software. The two anesthesia management schemes were sequentially coded in opaque cowhide envelopes by testing a list not involved in anesthesia management and attached to the case report. Before surgery, the anesthesiologist took the corresponding case report form in the randomization coding order and implemented the corresponding anesthesia management plan for the patients according to the randomization in the envelope. The sufentanil group (S group) received anesthesia induction with sufentanil, and the oxycodone group (O group) received anesthesia with oxycodone. All other procedures remained consistent and were kept confidential from both the postoperative follow-up personnel and the study subjects.

### 2.2 Sedative procedure

After the patient entered the operating room, vital signs were routinely monitored including heart rate, noninvasive cuff blood pressure, and pulse oxygen saturation. Additionally, the bispectral index (BIS**)** electrode was gently applied from the forehead to the left ear. A 16-gauge intravenous needle was used to establish peripheral intravenous access, and throughout the surgery, only sodium lactate Ringer's solution was used to supplement the blood volume at doses of 10–20 ml/kg/h. After using 100% O_2_ for 3 min, patients in the O group received 0.3 mg/kg of oxycodone, 2 mg/kg of propofol, and 0.6 mg/kg of rocuronium. Patients in the S group received 0.3 ug/kg of sufentanil, 2 mg/kg of propofol, and 0.6 mg/kg rocuronium. After the BIS value decreased to below 40, a laryngeal mask was placed, and mechanical ventilation was initiated in PCV-VG mode with a tidal volume of 6–8 ml/kg, inhalation of 50% O_2_, I:E ratio set to 1:2, and monitoring and maintenance of PETCO at 35–45 mmHg. The target-controlled infusion of propofol and remifentanil was maintained using total intravenous anesthesia. Propofol infusion was carried out in the Marsh mode, with an initial plasma target concentration of 1 ug/ml. Concentrations were adjusted to ±0.5 μg/ml to maintain the patient's BIS values fluctuating between 40 and 60 during the procedure. Noninvasive blood pressure and heart rate were recorded for each enrolled patient before anesthesia induction, immediately before laryngeal mask placement, immediately after laryngeal mask placement, immediately after pneumoperitoneum establishment, and immediately after removal of the laryngeal mask during the implementation of the anesthesia management protocol. After surgery, the patient was extubated and returned to the resuscitation room for further observation.

In both groups, pain at 0 h (t1), the abdominal wall incision, deep shoulder and back pain, and the degree of sedation, nausea, and vomiting were assessed. If the VAS score was 4, 2 mg of oxycodone was intravenously administered for rescue analgesia. If necessary, it was administered in 10 min intervals until a VAS score of 3 was achieved, and the amount of medication was recorded. At 0.5 h (t2), 2 h (t3), 2 h (t3), 6 h (t4), 6 h (t5), 8 h (t6) and 24 h (t7) postoperatively, the patient was followed-up by another anesthesiologist. The questions asked were the same as those at t1, and exhaust time, complications, and types were recorded. In both groups of patients, 4 ml of venous blood was drawn and placed in a red-capped tube without anticoagulant before the operation (t0) and at 0 h (t1), 6 h (t5), and 24 h (t7) postoperatively, before being centrifuged at 3,000 rpm for 15 min, and the supernatant was kept refrigerated at −80 °C. Serum levels of TNF-α, IL-6, and IL-10 in each enrolled patient were measured using enzyme-linked immunosorbent assays.

### 2.3 Outcome measures

The primary outcome measure was the degree of pain at seven pre-specified time points (0, 0.5, 2, 4, 6, 8, and 24 h postoperatively).

Secondary outcome measures included the visual analogue scale (VAS) score for analgesic effects, sedation degree (Ramsey), postoperative complications, and the serum levels of TNF-α, IL-6, and IL-10.

### 2.4 Statistical analysis

Statistical analyses were performed using SPSS Version 20 (IBM Corp., Armonk, New York, USA). Data analysis was based on the intention to treat. For continuous variables, descriptive statistics were calculated and reported as the mean ± standard deviation. Categorical variables are described using a frequency distribution. Student's *t*-test for paired samples was used to detect differences in the means of continuous variables, and the chi-square test was used in cases with low expected frequencies. Statistical significance was set at *p* < 0.05.

## 3 Results

### 3.1 Demographic data of patients

Demographic data including age, sex, BMI, and ASA grade, showed no statistically significant differences between the two groups (*p* > 0.05; Table 1).

**Table 1:** General conditions of the patients in both groups.

	Oxycodone (*n* = 30)	Sufentanil (*n* = 30)	*P*
Sex (male/female)	15/15	14/16	0.796
Age (years)	49.1 ± 12.0	46.7 ± 11.7	0.430
BMI (kg/m^2^)	24.7 ± 3.2	23.3 ± 3.1	0.092
ASA (I/II)	3/27	7/23	0.166

BMI, body mass index; ASA, American Society of Anesthesiologists physical status classification system.

### 3.2 Comparison of VAS scores for resting and active pain

Patients were followed up at 0 h (t1), 0.5 h (t2), 2 h (t3), 4 h (t4), 6 h (t5), and 8 h (t7) postoperatively. The VAS score of the patients’ subjective pain at 0.5 h was the same as the VAS score within 24 h (*p* > 0.05). In both groups, incision pain was not obvious after postoperative activity, and the VAS score for pain was assessed at 0.5 h postoperatively; however, there were no significant differences in pain VAS scores within 24 h postoperatively (*p* > 0.05; Table 2).

**Table 2:** VAS scores of wound, visceral, and shoulder pain at different time points (interquartile spacing).

	Group	Time points						
		t1	t2	t3	t4	t5	t6	t7
Resting wound pain	Oxycodone	0.0 (1) .0	0.5 (1) .0	0.0 (0) .0	0.0 (0) .0	0.0 (0) .0	0.0 (0) .0	.00 (0) .0
	Sufentanil	0.0 (1) .0	.01 (1) .0	.01 (0) .0	0.0 (0) .0	0.0 (0) .0	0.0 (0) .0	0.0 (0) .0
	*P*	0.599	0.807	0.269	0.232	0.690	0.690	1.000
Active wound pain	Oxycodone	.01 (1) .3	1.0 (2) .0	0.0 (0) .0	0.0 (0) .0	0.0 (0) .0	0.0 (0) .0	0.0 (0) .0
	Sufentanil	.01 (2) .0	1.0 (0) .3	0.0 (0) .0	0.0 (1) .0	0.0 (0) .0	0.0 (0) .0	0.0 (0) .0
	*P*	0.752	0.873	0.426	0.104	1.000	0.690	1.000
Resting visceral pain	Oxycodone	0.0 (1) .0	0.0 (1) .0	0.0 (1) .0	0.0 (0) .0	0.0 (0) .0	0.0 (0) .0	0.0 (0) .0
	Sufentanil	1.0 (0) .3	1.0 (1) .0	1.0 (1) .0	2.0 (0) .0	.02 (1) .0	1.0 (0) .0	.01 (0) .3
	*P*	<0.001	<0.001	<0.001	<0.001	<0.001	<0.001	<0.001
Active visceral pain	Oxycodone	0.0 (1) .0	0.0 (1) .3	0.0 (0) .0	0.0 (0) .0	0.0 (0) .0	0.0 (0) .0	0.0 (0) .0
	Sufentanil	2.0 (2) .3	.03 (2) .0	.03 (0) .3	2.5 (1) .0	2.0 (1) .0	.02 (1) .0	1.0 (1) .0
	*P*	<0.001	<0.001	<0.001	<0.001	<0.001	<0.001	<0.001
Resting shoulder pain	Oxycodone	0.0 (0) .0	0.0 (0) .0	0.0 (0) .0	0.0 (0) .0	0.0 (0) .0	0.0 (0) .0	0.0 (0) .0
	Sufentanil	0.0 (0) .0	0.0 (0) .0	0.0 (0) .0	0.0 (0) .0	0.0 (0) .0	0.0 (0) .0	0.0 (0) .0
	*P*	0.317	0.317	–	–	–	–	–
Active shoulder pain	Oxycodone	0.0 (0) .0	0.0 (0) .0	0.0 (0) .0	0.0 (0) .0	0.0 (0) .0	0.0 (0) .0	0.0 (0) .0
	Sufentanil	0.0 (0) .0	0.0 (0) .0	0.0 (0) .0	0.0 (0) .0	0.0 (0) .0	0.0 (0) .0	0.0 (0) .0
	*P*	0.317	0.317	–	–	–	0.317	–

VAS, visual analogue scale.

Compared with the S group, patients in the O group had lower VAS scores within 24 h postoperatively (*p* < 0.001); however, there was no statistical difference between the wound and shoulder pain scores (*p* > 0.05; Table 2).

### 3.3 Comparison of intraoperative and postoperative conditions

Intraoperative and postoperative conditions were recorded including pneumoperitoneum time, pneumoperitoneum pressure, surgical duration, duration of anesthesia, recovery time, intraoperative hypertension, hypotension, tachycardia, bradycardia, intraoperative relief analgesia, nausea and vomiting, and drainage tube placement. The results showed that the postoperative recovery time and extubation time in the O group was significantly shorter than that in the S group (*p* < 0.001). The postoperative relief analgesia was significantly better in the O group than in the S group (*p* < 0.01; Table 3).

**Table 3:** Statistics of intraoperative and postoperative conditions of patients in the two groups.

	Oxycodone (*n* = 30)	Sufentanil (*n* = 30)	*P*
Time of pneumoperitoneum (min)	81.6 ± 25.7	79.0 ± 25.8	0.697
Time of surgery (min)	95.8 ± 26.7	95.6 ± 31.9	0.979
Anesthesia time (min)	113.9 ± 29.1	111.7 ± 35.4	0.794
Wake-up time (min)	3.6 ± 2.4	8.2 ± 3.9	0.000
Extubation time (min)	2.4 ± 1.0	4.6 ± 1.4	0.000
Intraoperative hypertension (with/without)	0/30	2/28	0.150
Intraoperative hypotension (with/without)	0/30	1/29	0.313
Intra-operative tachycardia (with/without)	0/30	0/30	1.000
Slow movement of the operative center (with/without)	0/30	2/28	0.150
Remedial analgesia (with/without)	0/30	7/23	0.005
Nausea and vomiting (with/without)	4/26	1/29	0.161
Place the drainage (with/without)	0/30	0/30	0.313

### 3.4 Comparison of ramsay scores at different postoperative time points

Patients were followed up at 0 h (t1), 0.5 h (t2), 2 h (t3), 4 h (t4), 6 h (t5), 8 h (t6) and 24 h (t7). The Ramsay method was used to evaluate the postoperative sedation effect in the O group and the S group, and both treatments achieved good sedation effects after surgery according to the Ramsay score. Compared with the patients in Group S, the Ramsay score of patients in the O group decreased at 0 h (t1) (*p* < 0.001); however, the Ramsey score at the remaining time points did not significantly differ (Table 4).

**Table 4:** Ramsay scores at all postoperative times in both groups.

Group	Time points						
	t1	t2	t3	t4	t5	t6	t7
Oxycodone	2.0 (0.0)	2.0 (0.0)	2.0 (0.0)	2.0 (0.0)	2.0 (0.0)	2.0 (0.0)	2.0 (0.0)
Sufentanil	3.0 (1.0)	2.0 (0.0)	2.0 (0.0)	2.0 (0.0)	2.0 (0.0)	2.0 (0.0)	2.0 (0.0)
*P*	<0.001	0.150	1.000	1.000	1.000	1.000	1.000

Values are shown as median (interquartile range).

### 3.5 Determination of serum levels of inflammatory factors at different times in the two groups

A 4 ml venous blood sample was collected at baseline (t0), and the levels of TNF-α, IL-6, and IL-10 were measured at 0 h (t1), 6 h (t5) and 24 h (t7). The TNF-α level at 6 h in both groups was comparable, and serum TNF-α in the O group at 24 h was lower than that in the S group (*p* < 0.01). The amount of IL-6 in the serum of surgical patients was lower at 24 h in the O group than in the S group (*p* < 0.001).

Compared with preoperative levels, both treatments exhibited an increase in IL-10. At 6 h after surgery, IL-10 was increased in group S compared to that in group O (*p* < 0.001). After 24 h, the level of IL-10 increased in both groups, while group O showed higher levels than that in the S group (*p* < 0.01; Table 5).

**Table 5:** Changes in serum TNF-α, IL-6, and IL-10 levels.

	Group	Time points			
		t0	t1	t5	t7
TNF-α	Oxycodone (pg/L)	16.678 (2.847)	16.782 (3.413)	16.021 (4.568)	16.769 (3.413)*
	Sufentanil (pg/L)	19.161 (4.631)	18.037 (3.051)	18.199 (5.110)	22.358 (6.755)
	*P*	0.308	0.690	0.214	0.008
IL-6	Oxycodone (mg/L)	5.068 (3.060)	5.747 (6.264)	14.609 (9.143)	9.765 (7.061)*
	Sufentanil (mg/L)	5.347 (2.285)	6.529 (2.853)	16.956 (6.223)	20.317 (14.471)
	*P*	0.807	0.712	0.117	<0.001
IL-10	Oxycodone (ng/L)	9.077 (4.024)	10.186 (8.769)	14.583 (9.938)	15.335 (9.397*
	Sufentanil (ng/L)	10.004 (4.563)	11.377 (5.034)	9.167 (3.771)	9.714 (3.511)
	*P*	0.287	0.877	0.006	0.006

Values are shown as median (interquartile range).

*P < 0.05, as compared to other time points.

## 4 Discussion

In this study, oxycodone anesthesia induction showed better postoperative analgesia and a more reduced inflammatory response in patients undergoing laparoscopic cholecystolithotomy than sufentanil.

Laparoscopic cholecystostomy is a current surgical method for gallstones that retains the advantages of the gallbladder and its function (1). However, the puncture hole of the abdominal wall and the traction of the viscera induced pain and stress, reaching a VAS score of 4 points postoperatively. The severity of stress is related to the intracellular inflammatory response and the release of inflammatory factors, which have a significant impact on postoperative rehabilitation. Advanced analgesia is one of the most widely used concepts in anesthesia clinical work and refers to preventive measures actively taken before the generation of harmful stimulation. The types of analgesia mainly include suppressing the sensitivity of the central and peripheral nerves to stimulation, reducing acute stress and inflammatory responses, and minimizing the degree of pain (10). Visceral pain is characterized by inaccurate localization, diverse clinical symptoms, and no correlation between pain intensity and the degree of visceral injury (11). To relieve visceral pain, *μ* receptor agonists such as sufentanil and fentanyl are predominantly employed. However, their use is associated with elevated side effects such as respiratory depression, hypotension, postoperative nausea and vomiting, and pruritus. In addition, high doses of opioids largely cause hyperalgesia and acute tolerance several hours after administration, and these side effects greatly limit the use of *μ* receptor agonists.

As a dual receptor agonist of *μ* and *κ* opioid receptors, oxycodone has been proven to be more effective in relieving visceral pain. Compared to morphine, oxycodone has a shorter duration of action and a stronger analgesic effect. Most importantly, the same analgesic effect can be achieved with a smaller dose (12). A clinical study found that morphine and oxycodone exhibited comparable efficacy in addressing skin and muscle pain, whereas oxycodone provided better analgesia for esophageal pain caused by thermal and electrical stimulation (13). In terms of acute pain efficacy, oxycodone is administered at doses less than morphine 2 h after abdominal surgery because it can rapidly cross the blood-brain barrier into the brain.

The results of this study show that the average postoperative pain VAS scores at the bedside follow-up for both the O and S groups were below 3 points. Upon comparing the follow-up data between the two groups, it was observed that the O group exhibited significantly reduced postoperative resting and deep pain compared to the S group. However, there were significantly reduced, and the two groups of resting and activity after incision pain and shoulder pain were not significantly different. To some extent, the analgesic effect of oxycodone was better than that of sufentanil. Some previous studies have collected and analyzed the postoperative VAS score and the number of controlled analgesia presses of surgical patients and compared the analgesic effects of oxycodone and morphine in abdominal surgery. Their results showed that the VAS score and controlled analgesia presses in the oxycodone group were lower than those in the morphine group and that the analgesic effect of oxycodone after abdominal surgery was better than that of morphine (14). In this study, the patients found that the hemodynamic fluctuations associated with the immediate laryngeal mask, immediate establishment of the pneumoperitoneum, and immediate removal of the laryngeal mask were significantly better than those in the sufentanil group, and that oxycodone effectively maintained intraoperative hemodynamic stability. This study also found that the postoperative recovery and extubation times of patients in the oxycodone group were shorter than those in the sufentanil group, increasing patient comfort and improving the safety and reliability of the application of clinical anesthesia.

In a study of 60 patients undergoing gynecological laparoscopic surgery, Chen et al. (15) compared the postoperative intravenous control analgesic effects of oxycodone and sufentanil. The observation indices included the numeric rating scale scores and the number of effective presses on the analgesic pump. The results indicated that oxycodone was more satisfactory than sufentanil after gynecological laparoscopy, especially for relieving postoperative visceral pain. Although the type of surgery included in the above experiments was not the same as that in this study, the conclusion was that oxycodone demonstrates superior efficacy in relieving visceral pain compared to sufentanil. Additionally, Party Sha Jie et al. conducted a randomized clinical experiment involving 68 patients and compared the postoperative efficacy and safety of oxycodone and sufentanil for cervical cancer general anesthesia using Ramsay and NRS scores, which showed that oxycodone and sufentanil can provide satisfactory analgesia after general anesthesia, and the oxycodone analgesia time was longer than that of sufentanil (16). This study verified that the analgesic duration of oxycodone was longer than that of sufentanil. In a randomized clinical control trial of 60 patients with facial nerve microvascular decompression, Rui et al. (17) evaluated the anesthetic effect of oxycodone and sufentanil and recorded the VAS scores and related clinical adverse reactions. The results showed that oxycodone had a better postoperative analgesic effect. The above studies compared the effects of oxycodone and sufentanil during different surgical types, different types of analgesia, postoperative analgesia duration, and postoperative adverse effects, with conclusions similar to this study. Other studies demonstrated that the analgesic efficacy of oxycodone plus sufentanil is better than that of oxycodone alone or sufentanil after laparoscopic cholecystectomy (17). Moreover, compound anesthesia is emerging as a favored approach in clinical anesthesia, offering the advantages of enhanced analgesic effects and diminished anesthesia-related side effects. This trend provides novel avenues for future research in the field.

In terms of the levels of inflammatory factors, the results of this study found that serum levels of IL-6 and TNF-α decreased in surgical patients in the oxycodone group compared with the sufentanil group, indicating that oxycodone may be better than sufentanil in controlling inflammatory response. Gong et al. (18) evaluated the effect of nalbuphine on postoperative inflammatory responses, which showed that the serum levels of inflammation-related factors IL-6, TNF-α, and IL-1 were significantly lower than those in the sufentanil group and the incidence of adverse reactions was significantly reduced, indicating that nalbuphine could promote anesthetic and analgesic effects by inhibiting the release of some inflammatory factors. As a proinflammatory factor, IL-6 is a marker of surgical tissue injury and can be used as a measure of the degree of surgical trauma. Moreover, elevated levels of IL-6 serve as a sensitive marker of early trauma; therefore, inhibiting the release of IL-6 has a positive effect on postoperative rehabilitation. IL-6 is also an important mediator of postoperative pain. Research has evaluated the effect of three different anesthesia regimens, piperidine, norpivacaine, ropivacaine, and sufentanil + haloperidol, on the release of IL-6 and IL-10, as well as their influence on postoperative VAS scores. It was found that both treatments could achieve good anesthetic effects, and patients' serum inflammatory factors were significantly reduced (19). IL-10 has also been shown to exert anti-inflammatory effects by stimulating both immune and non-immune cells, and its overexpression of IL-10 can significantly reduce the inflammatory response in damaged skin (20). IL-10 has also been shown to exert anti-inflammatory effects by inhibiting the expression of IL-6 and other proinflammatory factors (21).

The subjects of this study were patients undergoing laparoscopic gallbladder-preserving cholecystolithotomy. It is well known that gallbladder-preserving surgery is less frequently performed worldwide. No similar study data can be used in published studies, so it is difficult to calculate the sample size accurately. As the largest gallbladder preservation surgery center in China, we innovatively focused on the perioperative pain problems of such patients and empirically designed it as a small sample randomized controlled clinical trial. This was a single-center clinical randomized controlled study with a limited sample size, which may have affected the results. Therefore, a prospective randomized controlled trial with a large multicenter sample size is required to validate the results of this study.

In conclusion, compared to sufentanil, oxycodone anesthesia induction showed better postoperative analgesia and a more reduced inflammatory response in patients undergoing laparoscopic cholecystolithotomy.

## Data Availability

The original contributions presented in the study are included in the article/Supplementary Material, further inquiries can be directed to the corresponding author.
